# Six Whole-Genome Assemblies of Yersinia pestis subsp. microtus bv. ulegeica (Phylogroup 0.PE5) Strains Isolated from Mongolian Natural Plague Foci

**DOI:** 10.1128/genomeA.00536-18

**Published:** 2018-06-21

**Authors:** Angelina A. Kislichkina, Alexandr G. Bogun, Lidiya A. Kadnikova, Nadezhda V. Maiskaya, Viktor I. Solomentsev, Angelika A. Sizova, Svetlana V. Dentovskaya, Sergey V. Balakhonov, Andrey P. Anisimov

**Affiliations:** a State Research Center for Applied Microbiology and Biotechnology, Obolensk, Russia; b Irkutsk Antiplague Research Institute of Siberia and the Far East, Irkutsk, Russia

## Abstract

Here, we report the draft genome sequences of six Yersinia pestis subsp. microtus bv. ulegeica strains isolated from the territory of Mongolia and representing the 0.PE5 phylogroup circulating in populations of voles and picas.

## GENOME ANNOUNCEMENT

Plague caused by Yersinia pestis is a zoonotic infection with an extraordinary epidemic potential. Although, genetically, Y. pestis is a monomorphic clone of its more diverse parental species, Yersinia pseudotuberculosis, there are several phylogenetic lines of the plague bacterium (1). The majority of Y. pestis strains have universal virulence against a wide range of mammals, including humans, while selective virulence is characteristic for strains from the 0.PE group circulating in populations of different Microtus spp. Such strains are highly virulent against their natural hosts and mice but have low virulence or are avirulent against guinea pigs and humans (2). The data of multilocus variable-number tandem-repeat analysis (MLVA) and clustered regularly interspaced short palindromic repeat (CRISPR) typing of Y. pestis subsp. microtus strains (3, 4) suggest that bv. ulegeica (0.PE5) strains from Mongolia represent the most recent guinea pig/human-avirulent branch of the Y. pestis phylogenetic tree. To date, only three whole-genome sequences of the strains from these ancient plague foci, which are characterized by a polymorphism of their circulating strains, have been deposited in GenBank (accession no. LIYO00000000, LIZG00000000, and LIZD00000000) (5). In this study, we sequenced eight additional strains isolated from the Sailugem (M01), Bukhen (M02), and Ochotona sp. Gurvan-Saikhan (M13) natural plague foci in Mongolia.

Whole-genome sequencing was performed using an Illumina MiSeq instrument according to the manufacturer’s instruction. DNA libraries were prepared using a Nextera DNA library preparation kit. A MiSeq v3 reagent kit was used for sequencing. For each genome, reads were assembled *de novo* using SPAdes v3.9 (http://cab.spbu.ru/software/spades/). Finally, we obtained from 167 to 180 contigs for each genome (Table 1). The genome sizes ranged from 4.58 to 4.69 Mb. Each genome contains 4,539 to 4,368 genes. Three strains bear three plasmids (pMT, pCD, and pPCP). Two strains lost pCD, and one strain lost pMT.

**TABLE 1:** Strain-identifying information and basic statistics on assemblies and annotations

Strain name	Focus^*a*^	SRA accession no.	GenBank assembly accession no.	Size (bp)	No. of contigs	Total genes	Coding genes	Plasmid^*b*^		
								pMT/ pFra	pCD/ pYV	pPCP/ pPst
SCPM-O-B-6301 (I-2231)	M01	SRR6794312	PVLW00000000	4,578,646	175	4,368	4,090	−	+	+
SCPM-O-B-6212 (I-2238)	M01	SRR6794311	PVLX00000000	4,616,215	167	4,420	4,161	+	−	+
SCPM-O-B-6218 (I-3190)	M13	SRR6794314	PVLY00000000	4,680,847	180	4,502	4,224	+	+	+
SCPM-O-DNA-15 (I-2236)	M01	SRR6794313	PVLZ00000000	4,621,120	169	4,494	4,238	+	−	+
SCPM-O-DNA-16 (I-2422 pMT+)	M02	SRR6794316	PVMA00000000	4,686,426	180	4,539	4,262	+	+	+
SCPM-O-DNA-17 (I-2457)	M13	SRR6794315	PVMB00000000	4,679,672	180	4,535	4,259	+	+	+

a Focus numbers are indicated according to reference 1.

b −, not present; +, present.

Study of branches of the Y. pestis tree can give additional data about intraspecies microevolution of Y. pestis, its spread around the world, and the formation of novel natural plague foci. Perhaps new information about genome structures of representatives of different SNP types will help to answer the main question—how the enteropathogenic bacterium Yersinia pseudotuberculosis, with a fecal-oral route of transmission, gave rise to the superbug Y. pestis, the cause of infamous deadly pandemic infection.

### Accession number(s).

The GenBank accession numbers for these eight genome sequences are listed in Table 1.
